# Simultaneous Determination of 78 Compounds of *Rhodiola rosea* Extract by Supercritical CO_2_-Extraction and HPLC-ESI-MS/MS Spectrometry

**DOI:** 10.1155/2021/9957490

**Published:** 2021-07-06

**Authors:** Alexander M. Zakharenko, Mayya P. Razgonova, Konstantin S. Pikula, Kirill S. Golokhvast

**Affiliations:** ^1^N. I. Vavilov All-Russian Institute of Plant Genetic Resources, B. Morskaya 42-44, Saint-Petersburg 190000, Russia; ^2^Far Eastern Federal University, Sukhanova 8, Vladivostok 690950, Russia; ^3^Pacific Geographical Institute, Far Eastern Branch of the Russian Academy of Sciences, Radio 7, Vladivostok 690041, Russia; ^4^Siberian Federal Scientific Centre of Agrobiotechnology, Centralnaya,Presidium, Krasnoobsk 633501, Russia

## Abstract

The plant *Rhodiola rosea* L. of family *Crassulaceae* was extracted using the supercritical CO_2_-extraction method. Several experimental conditions were investigated in the pressure range of 200–500 bar, with the used volume of cosolvent ethanol in the amount of 1% in the liquid phase at a temperature in the range of 31–70°C. The most effective extraction conditions are pressure 350 bar and temperature 60°C. The extracts were analyzed by HPLC with MS/MS identification. 78 target analytes were isolated from *Rhodiola rosea* (Russia) using a series of column chromatography and mass spectrometry experiments. The results of the analysis showed a spectrum of the main active ingredients *Rh. rosea*: salidroside, rhodiolosides (B and C), rhodiosin, luteolin, catechin, quercetin, quercitrin, herbacetin, sacranoside A, vimalin, and others. In addition to the reported metabolites, 29 metabolites were newly annotated in *Rh. rosea.* There were flavonols: dihydroquercetin, acacetin, mearnsetin, and taxifolin-O-pentoside; flavones: apigenin-O-hexoside derivative, tricetin trimethyl ether 7-O-hexosyl-hexoside, tricin 7-O-glucoronyl-O-hexoside, tricin O-pentoside, and tricin-O-dihexoside; flavanones: eriodictyol-7-O-glucoside; flavan-3-ols: gallocatechin, hydroxycinnamic acid caffeoylmalic acid, and di-O-caffeoylquinic acid; coumarins: esculetin; esculin: fraxin; and lignans: hinokinin, pinoresinol, L-ascorbic acid, glucaric acid, palmitic acid, and linolenic acid. The results of supercritical CO_2_-extraction from roots and rhizomes of *Rh. rosea*, in particular, indicate that the extract contained all biologically active components of the plant, as well as inert mixtures of extracted compositions.

## 1. Introduction

The plant *Rhodiola rosea* L. of family *Crassulaceae* is widely used in traditional medicine and traditional medical systems (Tibetan, Chinese, and Korean). Rhizomes and plant roots are mainly used for the preparation of medicinal products [1, 2].

The plant has an established popular name “golden root.” The name is determined not only by the color of the rhizome but also by its high price. The main medicinal raw material of *Rh. rosea* is rhizomes with roots, which are harvested from the end of flowering until the completion of the plant's vegetation. *Rh. rosea* grows in the mountains in the north of the European part of Russia, Siberia, the Urals, the mountains of Altai, the Tien Shan and the Far East, the mountains of Western Europe, Scandinavia, Mongolia, and on the spurs of the Himalayas. Brush wood of *Rh. rosea* is located at an altitude of 1700–2200 m above sea level. Since about the 80s, *Rh. rosea* has been one of the main adaptogenic plants and competes with such well-known adaptogens such as *Panax ginseng* and *Eleutherococcus*. Adaptogens are a pharmacological group of drugs of natural or synthetic origin, which can increase the body's resistance to various adverse environmental conditions [3–5].

*Rh. rosea* roots and rhizomes contain organic acids (citric, malic, oxalic, and succinic acid) and sugars (fructose, sucrose, glucose, sedoheptulose, essential oil, phenolic compounds, monoterpenes, sterols, cinnamon alcohol, and manganese) [6–8].

The active biologically active substances of *Rh. rosea* are tyrosol, salidroside, caffeic acid, gallic acid, methyl gallate, flavonoids (astragalin, kaempferol, rhodionine, rhodiosin, rhodiolinin, and rhodiolgin), and tannins of the pyrogallol group (Table 1). Monoterpenes are represented by rosiridol and its glycoside rosiridin, and sterols are represented by *β*-sitosterol and daucosterol. Cinnamon glycosides—rosin, rosarin, and rosavin—were isolated from the roots of *Rh. rosea* [9].

Information on the content of salidroside and rosavin in *Rh. rosea* is numerous and contradictory [10, 11]; Zang et al., 2019). Researchers still have not come to a consensus on the localization and activity of specialized biosyntheses, the nature of seasonal changes in glycoside content, and the variability in the accumulation of these substances in wild and cultivated plants [12–14].

Detailed comparative studies of the content of salidroside and rosavin in the organs of wild-growing and cultivated plants were carried out. Performed using a unified determination method showed the presence of glycosides only in the roots and caudex. The presence of rosavin and salidroside in the aerial organs (stems, leaves, inflorescences, and seeds) was not detected in any case [15].

Plants from different places of growth differed significantly in the accumulation of individual glycosides. The content of salidroside in the plant caudex varied from 9 to 20 mg/g dry weight. The largest accumulation of this glycoside was characterized by plants growing on rocks on the coast of the Barents Sea (Norway), as well as Ural plants growing on outcrops of bedrock with an insignificant soil layer. The minimum salidroside content was found in Altai plants. The highest content of rosavin (32 mg/g) was found in the caudex of plants of the subalpine ecotope in the Polar Urals, the lowest (10–12 mg/g) being in plants growing on the islands and the coast of the Barents Sea. Cultivated plants were not inferior for accumulation of rosavin to wild plants.

Differences in the accumulation of glycosides by plants of various ecotopes were revealed. So, in the Subpolar Urals, in the caudex of plants growing in faults and on ledges of rocks, more salidroside accumulates, but these plants were characterized by a low content of rosavin, 1.5–2 times less than in plants of the subalpine ecotope [15].

Cinnamic glycosides, and in particular rosavin, are believed to be the hallmark of the chemotaxonomic trait of *Rh. rosea* [16, 17]. Recently, however, literature has reported that this glycoside is present in other species of the genus *Rhodiola L*. The results confirmed the presence of rosavin in the caudex of *Rh. iremelica Boriss*. The concentration of salidroside and rosavin in the plant caudex was 7.1 ± 2.4 and 15.3 ± 2.9 mg/g, respectively. In the underground part of *Rh. quadrifida (Pall.) Fisch.* et Mey, rosavin was not detected, and the content of salidroside was about 10 mg/g dry weight [15].

In official medical practice, *Rh. rosea* root extract is intended for oral administration as a tonic and immunomodulating therapeutic agent. In the study of alcoholic extracts of *Rh. rosea*, their hepatoprotective, nootropic, cardioprotective, and antiarrhythmic properties were clearly demonstrated [18–20].

Cinnamic glycosides, also called cinnamyl glycosides and salidroside, are the main carriers of the biological activity of *Rh. rosea*, causing a positive pharmacological effect. With the presence of rosavin, rosin, and rosarin, many researchers attribute the increased biological activity of extracts of *Rh. rosea*, compared with drugs from other species of *Rhodiola*. Studies have shown the stimulating effect of drugs on the central nervous system. Of great interest is the ability of *Rh. rosea* to increase the body's resistance to the effects of various stress factors [21, 22]. *Rh. rosea* extract has immune stimulating, hepatoprotective, and antimicrobial effects [23, 24]. Studies have also been conducted on the antitumor effect of *Rh. rosea* extract [25–27].

This study considers the effectiveness of supercritical CO_2_-extraction of biologically active substances from roots and rhizomes of *Rh. rosea*. Previously, the authors of this article successfully used supercritical CO_2_ extraction to obtain biologically active substances from plants of the Far Eastern taiga *Panax ginseng*, *Rhododendron adamsii*, *Schisandra chinensis*, and sea cucumber which are extremely popular in traditional medicine of Southeast Asia [28, 29].

Supercritical fluid extraction (SFE) has been used since 1960s to analyze food and pharmaceutical products, isolate biologically active substances, and determine lipid levels in food and levels of toxic substances. In addition, the products do not have residues of organic solvents, which occur with conventional extraction methods, and solvents can be toxic, for example, in the case of methanol and *n-*hexane. High selectivity, easy solvent removal from the final product, and the use of moderate temperatures in the extraction process are the main attractive factors of SFE, leading to a significant increase in research for use in the food and pharmaceutical sectors [30, 31].

In Sweden, an article was published in 2009 that examined the extraction of rosavin from the roots and rhizomes of *Rh. rosea* using supercritical CO_2_-extraction. In this case, water was selected as a modifier of supercritical extraction, which gave a synergistic effect on the extraction yield of rosavin [32]. In China, researchers used supercritical CO_2_-extraction with ethanol modifier [33]. The purpose of this study was to extract the maximum amount of salidroside from the roots of *Rh. rosea*. The extraction conditions were chosen so that the yield of salidroside during supercritical extraction was much higher than the yield of the product when using classical extraction using a Soxhlet apparatus.

The results of SC-CO_2_-extraction of from roots and rhizomes of *Rh. rosea*, in particular, indicate that when using this technology, the extract contained all biologically active components of the plant, as well as inert mixtures of extracted compositions.

## 2. Experimental

### 2.1. Materials

Ground, dried root of *Rh. rosea* was obtained from the area near Lake Baikal, Russia. All samples were morphologically authenticated according to the current standard of Russian Pharmacopeia [34]. The volume weighted mean diameter of the powder was found as 550 *μ*m, as determined by dynamic light scattering (Hydro 2000MU Malvern Instruments Ltd.).

### 2.2. Chemicals and Reagents

HPLC-grade acetonitrile was purchased from Fisher Scientific (Southborough, UK), and MS-grade formic acid was purchased from Sigma-Aldrich (Steinheim, Germany). Ultrapure water was prepared from Siemens Ultra-Clear water purification system (Siemens Water Technologies, Germany), and all other chemicals were analytical grade.

### 2.3. Supercritical Fluid Extraction

A supercritical fluid extraction system was Thar SFE-500F-2-FMC50 (Thar Technology Inc., Pittsburgh, PA, USA) which is used in supercritical extraction. CO_2_ was compressed to the required pressure using a supercritical extraction compressor (Thar SFC, USA). A hot casing string heated the extraction vessel; the temperature was regulated by a thermostat (±1°C). A metering valve controlled the pressure. Shredded *Rhodiola* roots (50 g) were wrapped in a filter paper, charged to a one-liter extractor, and extracted with supercritical CO_2_ compressed to a supercritical state at a liquid flow rate of 250 g/min. Seven SFE extracts were obtained under different pressure conditions (100–400 bar) and temperatures (31–70°C). Ethanol served as the cosolvent in all cases. The extracts were collected in a separator. The pressure and temperature of the supercritical CO_2_ were optimized experimentally to achieve the maximum yield of the product during extraction.

### 2.4. Liquid Chromatography

HPLC was performed using Shimadzu LC-20 Prominence HPLC (Shimadzu, Japan), equipped with an UV-sensor and a Shodex ODP-40 4E reverse phase column to perform the separation of multicomponent mixtures. The gradient elution program was as follows: 0.01–4 min, 100% A; 4–60 min, 100–25% A; and 60–75 min, 25–0% A; control washing 75–120 min 0% A. The entire HPLC analysis was done with a DAD detector at wavelengths of 230 *η*m and 330 *η*m; the temperature corresponded to 17°C. The injection volume was 1 ml.

### 2.5. Mass Spectrometry

MS analysis was performed on an ion trap amaZon SL (Bruker Daltoniks, Germany) equipped with an ESI source in the negative ion mode. The optimized parameters were obtained as follows: ionization source temperature, 70°C; gas flow, 4l/min; nebulizer gas (atomizer), 7.3 psi; capillary voltage, 4500 V; end plate bend voltage, 1500 V; fragmentary, 280 V; and collision energy, 60 eV. An ion trap was used in the scan range *m*/*z* 100–1.700 for MS and MS/MS. The capture rate was one spectrum/s for MS and two spectra/s for MS/MS. Data collection was controlled by Windows software for Bruker Daltoniks. All experiments were repeated three times. A two-stage ion separation mode (MS/MS mode) was implemented.

## 3. Results and Discussion

**Figure 1:**
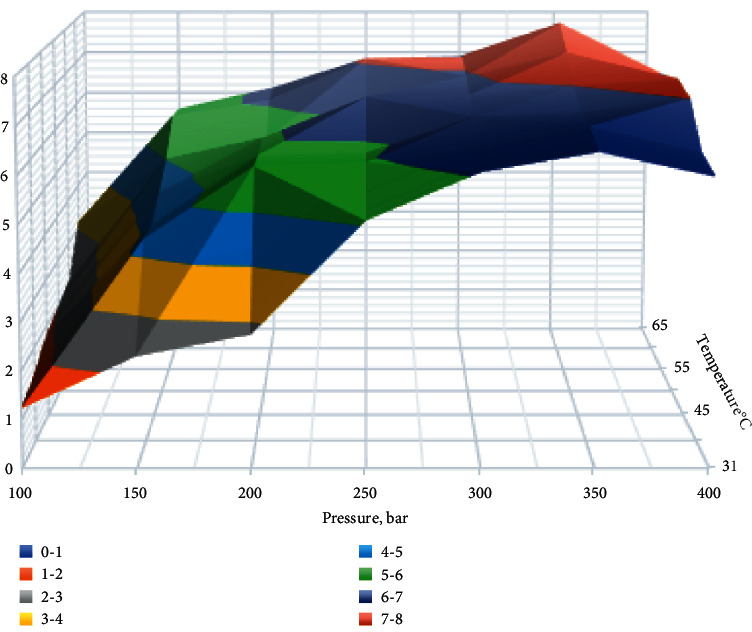
The effect of pressure and temperature on extraction efficiency of total yield of biologically active compounds (mg/g of extractable substance).

Several experimental conditions were investigated in the pressure range 200–500 bar, with the used volume of cosolvent ethanol in the amount of 1% in the liquid phase at a temperature ranging 31–70°C. Ethanol was used as the modifier due to its high solubility in CO_2_ and high polarity and ability to disturb solute-plant matrix bonding. As a result of using a wide range of pressures and temperatures empirically, the most efficient extraction conditions were found for extracting target analytes from the *Rh. rosea* roots. The most effective extraction conditions are pressure 350 bar and temperature 60°C (Figure 1).

Obtaining chemical profiles is an extremely important result in the biological analysis system. In this work, we used the HPLC-ESI-MS/MS method with additional ionization and analysis of fragmented ions. High accuracy mass spectrometric data were recorded on an ion trap amaZon SL (Bruker Daltoniks) equipped with an ESI source in the negative ion mode. The two-stage ion separation mode (MS/MS mode) was implemented.

**Figure 2:**
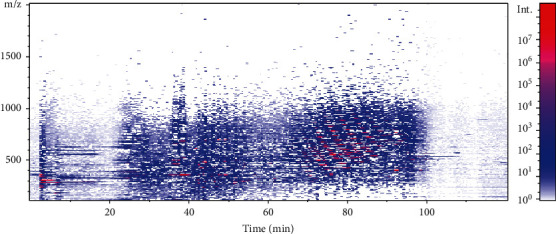
Distribution density of the analyzed chemical profiles in the ion chromatogram of *Rh. rosea* supercritical CO_2_-extract.

Figure 2 shows the distribution density of the analyzed chemical profiles in the ion chromatogram of the *Rh. rosea* supercritical CO_2_-extract, realized by mass spectrometry in the two-stage ion separation mode (MS/MS mode).

Visually, a rather high-density distribution of the target analytes in the analyzed extract was observed. All the chemical profiles of the samples were obtained by the HPLC-ESI-MS/MS method. A total of 300 peaks were detected in the chromatogram. By comparing the *m*/*z* values, the RT and the fragmentation patterns with the MS^2^ spectral data taken from the literature [2, 17, 35–50] or to search the data bases (MS2T, MassBank, HMDB). 78 metabolites were putatively identified as phenols, aromatic compounds, phenyl alkanoids, flavonoids, monoterpenoids, acyclic alcohol glycosides, anthocyanins etc. In addition to the reported metabolites, a number of metabolites were newly annotated in *Rh. rosea.*

A unifying system table consists of the molecular masses of the target analytes isolated from the supercritical CO_2_-extract of *Rh. rosea* for ease of identification (Table 2).

**Figure 3:**
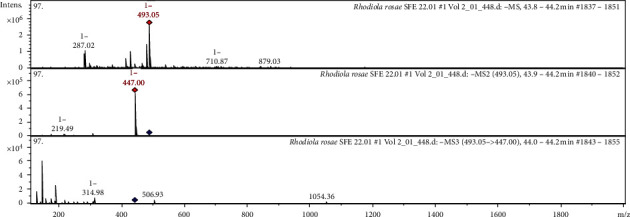
CID spectrum of the rhodioloside B from *Rh. rosea*, *m/z* 493.05.

The CID spectrum (collision induced dissociation spectrum) in negative ion modes of Rhodioloside B from *Rh. rosea* is shown in Figure 3.

The [M−H]^−^ ion produced two fragments with *m/z* 447.00 and *m/z* 219.49 **(**Figure 3***).*** The fragment ion with *m/z* 447.00 yields a daughter ion at *m/z* 314.98. The interpretation of the observed MS/MS spectra in comparison with those found in the literature was the main tool for putative identification of polyphenols. It was identified in the bibliography in extracts from *Rh. rosea* [50], from *Rhodiola crenulata* [35].

**Figure 4:**
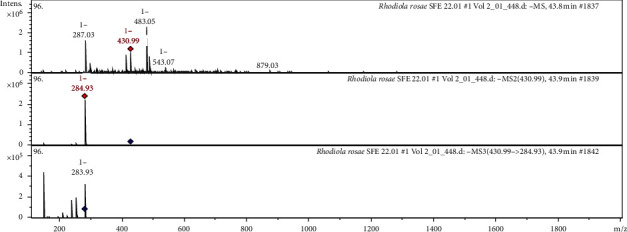
CID spectrum of luteolin-7-O-*α*-L-rhamnoside from *Rh. rosea*, *m/z* 430.99.

The CID spectrum in the negative ion mode of luteolin-7-O-*α*-L-rhamnoside from *Rh. rosea* is shown in Figure 4.

**Figure 5:**
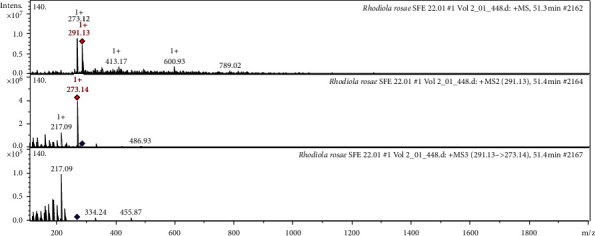
CID spectrum of catechin from *Rh. rosea*, *m/z* 291.13.

The [M−H]^−^ ion produced fragment with *m*/*z* 284.93 (Figure 5). The fragment ion with *m*/*z* 284.93 yields a daughter ion at m/*z* 283.93.

It was identified in the bibliography in extracts from *Rhodiola crenulata* [35]. The CID spectrum in the positive ion mode of catechin from *Rh. rosea* is shown in Figure 5. The [M+H]^+^ ion produced fragments with *m/z* 273.14 and *m/z* 217.09 (Figure 5). It was identified in the bibliography in extracts from *Rh. rosea* [50], from strawberry, cherimoya [36], and pear [45].

We isolated 78 target analytes from *Rhodiola rosea L*. (*Crassulaceae)* using a series of column chromatography and mass spectrometry experiments. The structures were elucidated using the data of stepwise fragmentation of ions during MS/MS spectrometry and compared with spectroscopic data in the literature. It is accepted that glycosides of cinnamon alcohol, and in particular Rosavin, are a distinctive chemotaxonomic sign of *Rh. rosea* [17]. However, lately, information has appeared in the literature on the presence of this glycoside in other species of the genus *Rhodiola L.* [15]. Thus, we can summarize the research that the supercritical extraction of the roots of *Rh. rosea* gives an extract that is extremely effective in terms of the composition of biologically active substances, which should find further application in both pharmacological, medical, and perfumery developments. In this regard, research on the development of a technology for obtaining supercritical drugs from rhizomes and roots of *Rh. rosea*, containing a complex of biologically active substances of this plant, and the development of modern drugs on their basis, presented primarily in the form of solid dosage forms, are relevant.

## 4. Conclusions

The *Rhodiola rosea* L. family *Crassulaceae* contains a large number of polyphenolic compounds and other biologically active substances. In this work, we tried to conduct a comparative metabolomic study of biologically active substances of *Rh. rosea* obtained from the area near Lake Baikal, Russia. HPLC in combination with a Bruker Daltoniks ion trap (tandem mass spectrometry) was used to identify target analytes in extracts.

The results showed the presence of 78 polyphenols and other compounds corresponding to the *Rhodiola rosea* family *Crassulaceae L*. species. In addition to the reported metabolites, 29 metabolites were newly annotated in *Rh. rosea.* There were flavonols: dihydroquercetin, acacetin, mearnsetin, and taxifolin-O-pentoside; flavones: apigenin-O-hexoside derivative, tricetin trimethyl ether 7-O-hexosyl-hexoside, tricin 7-O-glucoronyl-O-hexoside, and tricin O-pentoside and O-dihexoside; flavanone: eriodictyol-7-O-glucoside; flavan-3-ol gallocatechin; hydroxycinnamic acid; caffeoylmalic acid; di-O-caffeoylquinic acid; coumarins: esculetin; esculin, fraxin; lignans: hinokinin, pinoresinol, L-ascorbic acid, glucaric acid, palmitic acid, linolenic acid, etc.

The findings may support future research into the production of various pharmaceutical and dietary supplements containing *Rh. rosea* extracts. A wide variety of biologically active compounds opens up rich opportunities for the creation of new drugs and biologically active additives based on extracts from family *Crassulaceae*.

## Figures and Tables

**Table 1:** Some of the main active compounds of *Rh. rosea*.

S. no.	Compounds	Structure
1	Chlorogenic acid: C_16_H_18_O_9_	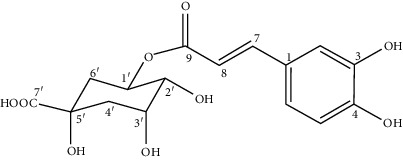
2	Rosiridin: C_16_H_28_O_7_	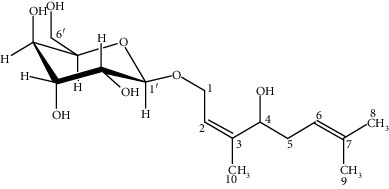
3	Rosavin: C_20_H_28_O_10_	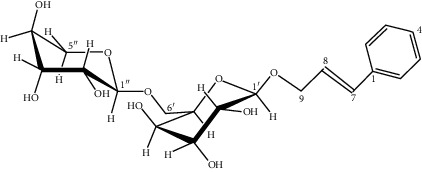
4	Salidroside: C_14_H_20_O_7_	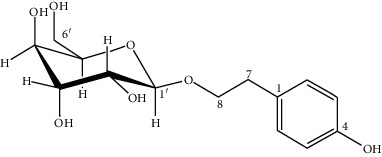
5	Rhodiolin (rhodiolinin): C_25_H_20_O_10_	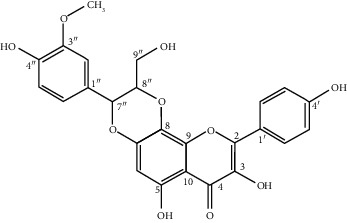

**Table 2:** Polyphenols and other substances identified from the SC-CO_2_ extracts of *Rh. rosea*.

No.	Compound group	Identification	Formula	Calculated mass	Observed mass [M-H]^−^	Observed mass [M+H]^+^	Observed mass [M+Na]^+^	MS/MS stage 1 fragmentation	MS/MS stage 2 fragmentation	References
Polyphenols										
1	Flavonol	Acacetin [linarigenin; buddleoflavonol]	C_16_H_12_O_5_	284.2635	—	285	—	240	212; 183; 165	*Mentha* [51]; *Ocimum* [41]

2	Flavonol	Kaempferol	C_15_H_10_O_6_	286.2363	—	287.11	—	269; 189; 133	213; 119	*Rhodiola sachalinensis* [52, 53]; *Rhodiola crenulata* [35, 54]; *Rhodiola sacra* [55]; *Impatiens glandulifera Royle* [56]

3	Flavonol	Quercetin	C_15_H_10_O_7_	302.2357	—	303.09	—	123; 147; 201; 233; 256	135; 175; 201	*Rhodiola rosea* [57]; *Rhodiola dumulosa* [58]; *Rhodiola crenulata* [35, 59]; *Impatiens glandulifera Royle* [56]*; Eucalyptus* [42]*; Triticum* [43]

4	Flavonol	Herbacetin (3, 5, 7, 8-tetrahydroxy-2-(4-hydroxyphenyl)-4H-chromen-4-one)	C_15_H_10_O_7_	302.2357	—	303.08	—	285	212; 268	*Rhodiola rosea* [3, 60–62]; *Rhodiola crenulata* [35]; *Ocimum* [41]

5	Flavonol	Dihydroquercetin (taxifolin; taxifoliol)	C_15_H_12_O_7_	304.2516	—	305.1	—	287; 269; 249; 231; 217; 147	269; 227; 213; 173; 161	*Larix dahurica* [63]; *Eucalyptus* [42]; *Vitis vinifera* [37]

6	Flavonol	Herbacetin 8-methyl ether	C_16_H_12_O_7_	316.2623	—	317.06	—	298; 183; 112	279; 228; 129	*Rhodiola crenulata* [35]; *Rhodiola dumulosa* [64]

7	Flavonol	Gossypetin (articulatidin; equisporol; 8-methoxy-hydroxyquercetin)	C_15_H_10_O_8_	318.2351	—	319.03	—	300.97	228; 166; 110	*Rhodiola rosea* [3, 62]

8	Flavonol	Mearnsetin	C_16_H_12_O_8_	332.2617	—	333.1	—	317; 292; 195	221; 183	*Eucalyptus* [42]

9	Flavonol	Rhodalin (herbacetin-8-O-beta-D-xylopyranoside)	C_20_H_18_O_11_	434.3503	—	434.96	—	389.90; 266.93	308; 345; 267; 167	*Rhodiola rosea* [17]

10	Flavonol	Taxifolin-O-pentoside	C_20_H_20_O_11_	436.371	—	436.99	—	391; 285; 177	352; 269; 173	*Vitis vinifera* [37]

11	Flavonol	Quercitrin (quercetin 3-L-rhamnoside; quercetrin)	C_21_H_20_O_11_	448.3769	—	448.90	—	302.95	169; 303	*Lotus japonicus* [65]; *Rhodiola rosea* [62]; *Rhodiola crenulata* [35, 59]

12	Flavonol	Rhodiolatuntoside	C_21_H_20_O_11_	448.3769	—	450.92	—	332.90	200.89; 154.87	*Rhodiola sachalinensis* [66]; *Rhodiola crenulata* [67]

13	Flavonol	Rhodiolinin (rhodiolin)	C_25_H_20_O_10_	480.4203	—	480.95	—	401; 313; 233; 173	357; 313; 269; 233; 145	*Rhodiola rosea* [2, 16]; *Rhodiola sachalinensis* [52, 53, 68]; *Rhodiola crenulata* [69]

14	Flavonole glycoside	Kaempferol-3-xylosyl-glycoside	C_26_H_28_O_15_	580.4915	—	581.09	—	331; 509; 469; 375; 243	330.89; 287.99; 141.74	*Rhodiola rosea* [61]

15	Flavonole glycoside	Rhodiosin	C_27_H_30_O_16_	610.5175	—	610.82	—	303; 449	169	*Rhodiola rosea* [2, 16, 70, 71]; *Rhodiola sachalinensis* [52, 68]; *Rhodiola crenulata* [69]

16	Flavonole glycoside	Rhodiolgidin	C_27_H_30_O_17_	626.5179	—	627.30	—	344.78	344.7	*Rhodiola rosea* [3, 17]; *Rhodiola crenulata* [35]

17	Flavan-3-ol	Catechin	C_15_H_14_O_6_	290.2681	—	291.97	—	250	227	*Rhodiola rosea* [50]; *Rhodiola crenulata* [35]; strawberry, cherimoya [36]; pear [45]

18	Flavan-3-ol	Epicatechin ((2R,3R)-2-(3,4-dihydroxyphenyl)-3,5,7-chromanetriol)	C_15_H_14_O_6_	290.2681	—	291.1	—	261; 273; 217; 173; 163	243; 191; 173; 143	*Rhodiola rosea* [50]; *Rhodiola crenulata* [35]; *Rhodiola kirilowii* [72]

19	Flavan-3-ol	Gallocatechin ((+)-gallocatechin)	C_15_H_14_O_7_	306.27	305.06	—	—	179; 168; 261	124	Red wine [73]; *Licania ridigna* [74]

20	Flavan-3-ol	(-)-Epicatechin gallate	C_22_H_18_O_10_	442.3723	—	443.01	—	363.12	319.16	*Rhodiola rosea* [39]; *Rhodiola crenulata* [35, 75]; *Rhodiola kirilowii* [50, 76]

21	Flavanone	Eriodictyol-7-O-glucoside (pyracanthoside; miscanthoside)	C_21_H_22_O_11_	450.3928	—	451.00	—	333; 433; 155	288; 201	*Impatiens glandulifera Royle* [56]

22	Flavone	Luteolin	C_15_H_10_O_6_	286.2363	285.02	—	—	241; 168; 124	124.02	*Rhodiola crenulata* [35, 54]; *Rhodiola kirilowii* [72]; *Rhodiola sachalinensis* [53, 77]

23	Flavone	Tricin	C_17_H_14_O_7_	330.2889	329.18	—	—	299; 311; 229; 171	211.04; 125.14	*Triticum aestivum L.* [77, 78]; *Rhodiola rosea* [61, 79]; *Rhodiola sacra* [55]; *Rhodiola sachalinensis* [53]; *Rhodiola crenulata* [59]

24	Flavone	Luteolin-7-O-*α*-L-rhamnoside	C_21_H_20_O_10_	432.3775	430.99	—	—	284.93	283.93	*Rhodiola crenulata* [35];

25	Flavone	Tricin 7-O-glucoside	C_23_H_24_O_12_	492.4295	—	493.11	—	401; 292; 201	383; 329; 280; 156	*Rhodiola rosea* [61, 70, 79]; *Rhodiola crenulata* [59]

26	Flavone	Apigenin-O-hexoside derivative	C_26_H_25_O_12_	529.4695	—	531.08	—	433; 485; 243; 177	399; 310	*Strawberry* [36]

27	Flavone	Tricetin trimethyl ether, 7-O-hexoside malonylated	C_27_H_28_O_15_	592.5022	591.23	—	—	533; 437; 323	197.01	*Triticum aestivum L.* [77]

28	Flavone	Tricin, 7-O-glucoronyl-O-hexoside	C_29_H_32_O_18_	668.5536	—	669.13	—	419; 375; 271	375; 243; 171	*Triticum aestivum L.* [77]

29	Flavone	Tricin trimethyl ether, 7-O-hexosyl-hexoside	C_30_H_36_O_17_	668.5966	—	669.01	—	419; 557; 331; 287	375; 331; 215	*Triticum aestivum L.* [77]

30	Flavone	Tricin, O-pentoside; O-dihexoside	C_35_H_44_O_21_	800.7113	—	801.24	—	409; 655; 509; 252	—	*Triticum aestivum L.* [77]

31	Hydroxycinammic acid	Ferulic acid	C_10_H_10_O_4_	194.184	—	195.07	—	176.8	—	*Rhodiola crenulata* [35]; *Triticum* [43]

32	Hydroxycinammic acid	Caffeoylmalic acid	C_13_H_12_O_8_	296.2296	—	297.09	—	279; 211; 163	265; 163; 135	Strawberry [36]

33	Cinnamate ester	4-O*-p*-Coumaroylquinic acid	C_16_H_18_O_8_	338.3098	—	338.94	—	189; 151	—	Pear [45]

34	Cinnamic alcohol glycoside)	Rosin (trans-cinnamyl O-beta-D-glycopyranoside)	C_15_H_20_O_6_	296.3157	—	297.06	—	255; 179; 115	215; 110	*Rhodiola rosea* [16, 49, 80]; *Rhodiola crenulata* [35]; *Rhodiola sachalinensis* [53]

35	Cinnamic alcohol glycoside	Triandrin	C_15_H_20_O_7_	312.3151	—	313.21	—	268.14	240; 211; 193	*Rhodiola crenulata* [35, 54]; *Rhodiola rosea* [10, 81]

36	Cinnamic alcohol glycoside	Sachaliside 1	C_15_H_20_O_7_	312.3151	311.13	—	—	309.08; 182.96	247.08; 119.01	*Rhodiola rosea* [9]

37	Cinnamic alcohol glycoside	*p*-Hydroxyphenacyl-*β*-D-glucopyranoside	C_14_H_18_O_8_	314.2879	—	314.97	—	294; 163	—	*Rhodiola crenulata* [35, 82];
38	Cinnamic alcohol glycoside	(2E)-3-(4-methoxyphenyl)-2-propen-1-yl-beta-D-glycopyranoside	C_16_H_22_O_7_	326.3417	325.09	—	—	182.99	119.09	*Rhodiola rosea* [9]

39	Cinnamic alcohol glycoside	Coniferin	C_16_H_22_O_8_	342.3411	—	343.01	—	240; 301; 129	240; 183	*Rhodiola crenulata* [35, 54]

40	Phenylpropanoid (cinnamicacid derivative glycoside)	Chlorogenic acid (3-O-caffeoylquinic acid)	C_16_H_18_O_9_	354.3087	—	355.04	—	335; 285; 203	200.0	*Rhodiola rosea* [2]; *Eucalyptus* [42]; *Triticum* [43];

41	Cinnamic alcohol glycoside	Rosavin (trans-cinnamil O-(6′-O-alpha-L-arabinopyranosyl-beta-D-glycopyranoside)	C_20_H_28_O_10_	428.4303	—	—	451.00	333; 155; 201	200.94	*Rhodiola rosea* [16, 49, 83]; *Rhodiola crenulata* [84]; *Rhodiola sachalinensis* [53]; *Rhodiola quadrifida* [2, 85]

42	Cinnamic alcohol glycoside	Rosarin (trans-cinnamyl O-(6′-O-alpha-L arabinofuranosyl-beta-D-glycopyranoside)	C_20_H_28_O_10_	428.4303	—	429.01	—	285; 199	384; 328; 230; 159	*Rhodiola rosea* [9, 16, 49, 83]; *Rhodiola sachalinensis* [53]

43	Phenylpropanoid (cinnamic acid derivative)	Di-O-caffeoylquinic acid	C_25_H_24_O_12_	516.4509	—	516.86	—	352; 431; 276	200; 135	*Pear* [45]

44	Gallic acid derivative	6-O-galloyl-salidroside	C_21_H_24_O_11_	452.4087	—	453.09	—	435; 209; 336	226; 336; 417	*Rhodiola crenulata* [35, 54]*; Rhodiola rosea* [39]

45	Gallic acid derivative	1,2,6-Tri-O-galloyl-beta-D-glucoside	C_27_H_24_O_18_	636.4687	—	637.28	—	507; 566; 620; 488; 366; 189	—	*Rhodiola rosea* [39]

46	Anthocyanidin	Pelargonidin-3-glucoside (callistephin)	C_21_H_21_ClO_10_	468.8444	—	469.88	—	357.05	247.00	*Triticum* [43]

47	Anthocyanidin	Pelargonidin (3-O-(6-O-malonyl-beta-D-glucoside))	C_24_H_23_O_13_	519.4388	—	520.10	—	433; 184	307; 163	*Gentiana lutea* [86]; wheat [87]

48	Proanthocyanidin	Proanthocyanidin B1 (procyanidin B1; procyanidin dimer B1)	C_30_H_26_O_12_	578.5202	577.21	579.07	—	197; 254; 351; 393; 407; 421	196.94; 133.04; 182.93	Pear [45]; *Eucalyptus* [42]

49	Anthocyanidin	Cyanidin-3-(3″,6″-dimalonylglucoside)	C_27_H_24_O_17_	620.4773	—	621.17	—	619; 432; 264	601; 518; 419	Wheat [87]

50	Anthocyanidin	Pelargonidin (3-O-(6-O-malonyl-beta-D-glucoside)-5-beta-D-glucoside	C_30_H_33_O_18_	681.5812	—	682.10	—	515.58; 353.14	351; 295; 173	*Gentiana lutea* [86]
51	Coumarin	Esculetin (cichorigenin; esculetin)	C_9_H_6_O_4_	178.1415	—	179.02	—	147.01	119.03	*Ledum palustre* [38]; *Vitis vinifera* [37]

52	Coumarin	Esculin (esculin; esculoside; polichrome)	C_15_H_16_O_9_	340.2821	—	340.91	—	133; 283; 322	175; 133	Dog plasma [38]*;* rat plasma [88]

53	Coumarin glucoside	Fraxin (Fraxetin-8-O-glucoside)	C_16_H_18_O_10_	370.3081	—	370.97	—	356; 193; 123	207.02	Dog plasma [38]; rat plasma [88]

54	Lignan	Hinokinin	C_20_H_18_O_6_	354.3533	—	355.01	—	337; 283; 203	239; 133	*Triticum aestivum L.* [89]; *Bursera simaruba* [90]

55	Lignan	Pinoresinol	C_20_H_22_O_6_	358.3851	—	359.02	—	341; 187	323; 187	*Triticum aestivum* L. [78]; *Eucommia cortex* [47]

56	Aryl-beta-glycoside	Arbutin	C_12_H_16_O_7_	272.2512	—	273.17	—	217; 163	161.09	Strawberry, blueberry, pear [91]; pear [45]

Others										
57	Natural water-soluble vitamin	L-ascorbic acid	C_6_H_8_O_6_	176.1241	—	176.98	—	145.00	117.03	Strawberry, lemon, papaya [36]

58	Aldaric acid	Glucaric acid (D-glucaric acid)	C_6_H_10_O_8_	210.1388	—	211.01	—	192; 115	129.05	Cherimoya, papaya [36]

59	Monobasic saturated carboxylic acid	Palmitic acid (hexadecanoic acid; palmitate)	C_16_H_32_O_2_	256.4241	—	257.02	—	237; 137	221; 125	*Salviae* [44]

60	Acyclic alcohol nitrile glycoside	Heterodendrin ((2R)-2-(*β*-D-glucopyranosyloxy)-3-methylbutanenitrile)	C_11_H_19_O_6_N	261.2717	—	263.96	—	155; 228	—	*Rhodiola crenulata* [35]

61	Monobasic saturated carboxylic acid	Linolenic acid (alpha-linolenic acid; linolenate)	C_18_H_30_O_2_	278.4296	—	279.1	—	261; 243; 187; 123	173; 131	*Salviae* [44]; rice [48]

62	Phenylethane glycoside	Picein (ameliaroside; salicinerin; salinigrin; piceoside)	C_14_H_18_O_7_	298.2901	—	299	—	271; 211; 179	254; 225; 197	*Rhodiola rose* [9]; *Rhodiola crenulata* [82]

63	Phenylethane glycoside	Salidroside (2-(4-hydroxyphenyl) ethyl *β*-D-glucopyranoside)	C_14_H_20_O_7_	300.3044	—	301.15	—	240; 201	183; 110	*Rhodiola crenulata* [35, 54]; *Rhodiola rosea* [1, 92, 93]*; Rhodiola sachalinensis* [53]; *Rhodiola kirilowii* [2]

64	Phenylethane glycoside	Icariside D2	C_14_H_20_O_7_	300.3044	—	301.06	—	240; 201; 135	183; 113	*Rhodiola rosea* [39]; *Rhodiola crenulata* [54, 82]; *Rhodiola sacra* [55];

65	Acyclic alcohol glycoside	Creoside II	C_14_H_26_O_7_	306.352	—	307.99	—	199; 255	—	*Rhodiola crenulata* [35, 54]
66	Phenylethane glycoside	Viridoside	C_15_H_22_O_7_	314.331	—	315.04	337.11	319.13; 209.08	151; 207; 262; 301	*Rhodiola viridula* [94]; *Rhodiola rosea* [83]; *Rhodiola crenulata* [35]; *Rhodiola sachalinensis* [53]

67	Acyclic alcohol glycoside	Rosiridin (3,7-dimethylocta-2,6-diene-1,4-diol; 1-O-beta-D-glucopyranoside)	C_16_H_28_O_7_	332.3893	—	333.02	—	247; 175	181.93	*Rhodiola crenulata* [35]*; Rhodiola rosea* [2, 17, 49]*; Rhodiola sachalinensis* [95]

68	Acyclic alcohol glycoside	Rhodioloside A	C_16_H_28_O_8_	348.3887	—	349.02	371.03	271; 281; 305; 331; 257; 231; 219; 167; 141	268; 256; 243; 229; 215; 193; 143	*Rhodiola rosea* [1, 92]; *Rhodiola crenulata* [35]

69	Acyclic alcohol glycoside	Rhodioloside D	C_16_H_30_O_8_	350.4046	—	351.06	—	258; 220; 131	257; 141	*Rhodiola rosea* [1, 83, 92]; *Rhodiola crenulata* [35]

70	Tetracyclic diterpenoid	Grayanotoxin II	C_20_H_32_O_5_	352.4651	—	353.04	—	335; 282; 203	315; 245; 113	Grayanotoxins [96]

72	Benzidine glycoside	Phenylmethyl (6-O-alpha-L-arabinopyranosyl-beta-D-glycopyranoside)	C_18_H_26_O_10_	402.3930	—	402.86	—	343; 283; 175	283	*Rhodiola rosea* [83]; *Rhodiola sachalinensis* [53]

73	Acyclic alcohol glycoside	Rhodiooctanoside	C_19_H_36_O_10_	424.4831	—	424.94	—	290.96	173; 261	*Rhodiola crenulata* [35, 54]; *Rhodiola kirilowii* [97]; *Rhodiola sacra* [98]

74	Phenylethane glycoside	Mongrhoside	C_20_H_30_O_11_	446.4456	—	446.65	—	243; 379; 311	174.84	*Rhodiola rosea* [83]

75	Acyclic alcohol glycoside	Creoside V	C_21_H_38_O_10_	450.5204	—	473.15	—	471; 254; 401	463.61	*Rhodiola crenulata* [35];

76	Hydroxy acid	Ursolic acid	C_30_H_48_O_3_	456.7003	—	457.17	—	412; 307	368; 269	*Ocimum* [41]; pear [45]

77	Acyclic alcohol glycoside	Rhodioloside E	C_21_H_38_O_11_	466.5198	—	467.95	—	399.94; 265; 332	331.88	*Rhodiola rosea* [1, 92]; *Rhodiola crenulata* [35, 54]; *Rhodiola sachalinensis* [13]; *Rhodiola sacra* [55]

78	Acyclic alcohol glycoside	Rhodioloside B	C_22_H_38_O_12_	494.5299	493.22	—	517.97	447; 220	314.98	*Rhodiola rosea* [1, 92]; *Rhodiola crenulata* [35]

## Data Availability

No data were used to support this study.

## References

[1] Saratikov A. S., Krasnov E. A., Chnikina L. A. (1968). Rhodiolosid, a new glycoside from *Rhodiola rosea* and its pharmacological properties. *Pharmazie*.

[2] Wiedenfeld H., Dumaa M., Malinowski M., Furmanowa M., Narantuya S. (2007). Phytochemical and analytical studies of extracts from *Rhodiola rosea* and *Rhodiola quadrifida*. *Die Pharmazie*.

[3] Zapesochnaya G. G., Kurkin V. A., Shchavlinskii A. N. (1985). Flavonoids of the above‐ground part of *Rhodiola rosea*. II. Structure of novel glycosides of herbacetin and gossypetin. *Chemistry of Natural Compounds*.

[4] Saratikov A. S., Krasnov E. A. (2004). *Rhodiola Rosea (Golden Root)*.

[5] Kurkin V. A. (2015). *Rhodiola Rosea (Golden Root): Drugs Production and Standardization: Monography*.

[6] Dubichev A. G., Kurkin V. A., Zapesochnaya G. G., Vorontsov E. D. (1991). Chemical composition of the rhizomes of the Rhodiola rosea by the HPLC method. *Chemistry of Natural Compounds*.

[7] Buchwald W., Mscisz A., Krajewska-Patan A., Furmanova M., Mielcarek S., Mrozikiewicz P. M. (2006). Contents of biologically active compounds in *Rhodiola rosea* roots during the vegetation period. *Herba Polonica*.

[8] Ma G., Li W., Dou D. (2006). Rhodiolosides A-E, monoterpene glycosides from Rhodiola rosea. *Chemical and Pharmaceutical Bulletin*.

[9] Tolonen A., Pakonen M., Hohtola A., Jalonen J. (2003). Phenylpropanoid glycosides from *Rhodiola rosea*. *Chemical and Pharmaceutical Bulletin*.

[10] Zapesochnaya G. G., Kurkin V. A., Boiko V. P., Kolkhir V. K. (1995). Phenylpropanoids as promising biologically active substances from medicinal plants. *Pharmaceutical Chemistry Journal*.

[11] Rodin I. A., Stavrianidi A. N., Braun A. V., Shpigin O. A., Popik M. V. (2012). Simultaneous determination of salidroside, rosavin, and rosarin in extracts from *Rhodiola rosea* by high performance liquid chromatography with tandem mass spectrometry detection. *Mass-Spektrometria.*.

[12] Ioset K. N., Nyberg N. T., van Diermen D. (2011). Metabolic profiling of Rhodiola rosea rhizomes by 1H NMR spectroscopy. *Phytochemical Analysis*.

[13] Li T., Zhang H. (2008). Identification and comparative determination of rhodionin in traditional Tibetan medicinal plants of fourteen Rhodiola species by high-performance liquid chromatography-photodiode array detection and electrospray ionization-mass spectrometry. *Chemical and Pharmaceutical Bulletin*.

[14] Evstatieva L., Todorova M., Antonova D., Staneva J. (2010). Chemical composition of the essential oils of *Rhodiola rosea* L. of three different origins. *Pharmacognosy Magazine*.

[15] Zakhozhi I. G. (2006). Physiological-biochemical bases of accumulation secondary metabolism products–salidroside and rosavin in plants of rhodiola rosea.

[16] Zapesochnaya G. G., Kurkin V. A. (1982). Glycosides of cinnamyl alcohol from the rhizomes of Rhodiola rosea. *Chemistry of Natural Compounds*.

[17] Kurkin V. A., Zapesochnaya G. G. (1985). *Chemical Composition and Pharmacological Characteristics of Rhodiola Rosea. J. Medicinal Plants*.

[18] Arbuzov A. G., Maslov L. N., Burkova V. N., Krylatov A. V., Konkovskaia I. N., Safronov S. M. (2009). Phytoadaptogens-induced phenomenon similar to ischemic preconditioning. *Rossiiskii Fiziologicheskii Zhurnal Imeni I.M. Sechenova*.

[19] Maslov L. N., Lishmanov I. B. (2007). Cardioprotective and antiarrhythmic properties of *Rhodiolae roseae* preparations. *Eksperimental’naia i Klinicheskaia Farmakologiia*.

[20] Wu T., Zhou H., Jin Z. (2009). Cardioprotection of salidroside from ischemia/reperfusion injury by increasing N-acetylglucosamine linkage to cellular proteins. *European Journal of Pharmacology*.

[21] Spasov A. A., Wikman G. K., Mandrikov V. B., Mironova I. A., Neumoin V. V. (2000). A double-blind, placebo-controlled pilot study of the stimulating and adaptogenic effect of *Rhodiola rosea* SHR-5 extract on the fatigue of students caused by stress during an examination period with a repeated low-dose regimen. *Phytomedicine*.

[22] Bystritsky A., Kerwin L., Feusner J. D. (2008). A pilot study of *Rhodiola rosea* (Rhodax) for generalized anxiety disorder (GAD). *The Journal of Alternative and Complementary Medicine*.

[23] Udintsev S. N., Krylova S. G., Fomina T. I. (1992). The enhancement of the efficacy of adriamycin by using hepatoprotectors of plant origin in metastases of Ehrlich’s adenocarcinoma to the liver in mice. *Voprosy Onkologii*.

[24] Zhang Y., Liu Y. (2005). Study on effects of salidroside on lipid peroxidation on oxidative stress in rat hepatic stellate cells. *Zhong Yao Cai*.

[25] Dement’eva L. A., Iaremenko K. V. (1987). Effect of a Rhodiola extract on the tumor process in an experiment. *Voprosy Onkologii*.

[26] Udintsev S. N., Shakhov V. P. (1991). The role of humoral factors of regenerating liver in the development of experimental tumors and the effect of Rhodiola rosea extract on this process. *Neoplasma*.

[27] Udintsev S. N., Schakhov V. P. (1991). Decrease of cyclophosphamide haematotoxicity by Rhodiola rosea root extract in mice with Ehrlich and Lewis transplantable tumours. *European Journal of Cancer and Clinical Oncology*.

[28] Zakharenko A., Romanchenko D., Thinh P. D. (2020). Features and advantages of supercritical СО_2_ extraction of sea cucumber *Cucumaria frondosa japonica* semper, 1868. *Molecules*.

[29] Razgonova M. P., Zakharenko A. M., Grudev V., Ercisli S., Golokhvast K. S. (2020). Comparative analysis of the multicomponent composition of far east Sikhotinsky Rhododendron (*Rh. sichotense*) and East Siberian Rhododendron (*Rh. adamsii*) using supercritical CO_2_-extraction and HPLC-MS/MS spectrometry. *Molecules*.

[30] Baldino L., Scognamiglio M., Reverchon E. (2020). Supercritical fluid technologies applied to the extraction of compounds of industrial interest from *Cannabis sativa* L. and to their pharmaceutical formulations: a review. *Journal of Supercritical Fluids*.

[31] Morozov Y. A., Pupykina K. A., Blagorazumnaya N. V., Aliev A. M., Morozova E. V. (2018). Comparative analysis of carbon dioxide extracts from plant material of Schisandra chinensis: leaves, woody stems, rhizomes with roots. *Bashkortostan Medical Journal*.

[32] Iheozor-Ejiofor P., Szwajcer Dey E. (2009). Extraction of rosavin from *Rhodiola rosea* root using supercritical carbon dioxide with water. *Journal of Supercritical Fluids*.

[33] Wu Y.-X., Wang Q., Liu B., You M.-Y., Jin T. (2011). Supercritical carbon dioxide extraction of salidroside from *Rhodiola rosea L var* rosea root. *Journal of the Chinese Chemical Society*.

[34] State Pharmacopeia XIV. 2018 [in Russ.]

[35] Han F., Li Y., Ma L. (2016). A rapid and sensitive UHPLC-FT-ICR MS/MS method for identification of chemical constituents in *Rhodiola crenulata* extract, rat plasma and rat brain after oral administration. *Talanta*.

[36] Spinola V., Pinto J., Castilho P. C. (2015). Identification and quantification of phenolic compounds of selected fruits from Madeira Island by HPLC-DAD-ESI-MSn and screening for their antioxidant activity. *Food Chemistry*.

[37] Goufo P., Singh R. K., Cortez I. (2020). Phytochemical a reference list of phenolic compounds (including stilbenes) in grapevine (Vitis vinifera L.) roots, woods, canes, stems, and leaves. *Antioxidants*.

[38] Wang Z., Zhu W., Liu H. (2018). Simultaneous determination of aesculin, aesculetin, fraxetin, fraxin and polydatin in beagle dog plasma by UPLC-ESI-MS/MS and its application in a pharmacokinetic study after oral administration extracts of *Ledum palustre* L. *Molecules*.

[39] Lee T. H., Hsu C. C., Hsiao G., Fang J. Y., Liu W. M., Lee C. K. (2016). Anti‐MMP‐2 activity and skin‐penetrating capability of the chemical constituents from *Rhodiola rosea*. *Planta Medica*.

[40] Fan W., Tezuka Y., Komatsu K., Namba T., Kadota S. (1999). Prolyl endopeptidase inhibitors from the underground part of *Rhodiola sacra* S. H. Fu. *Biological and Pharmaceutical Bulletin*.

[41] Pandey R., Kumar B. (2016). HPLC-OTOF-MS/MS-based rapid screening of phenolics and triterpenic acids in leaf extracts of *Ocimum* species and heir interspecies variation. *Journal of Liquid Chromatography & Related Technologies*.

[42] Santos S. A. O., Freire C. S. R., Domingues M. R. M., Silvestre A. J. D., Neto C. P. (2011). Characterization of phenolic components in polar extracts of *Eucalyptus globulus* labill. Bark be high-performance liquid chromatography-mass spectrometry. *Journal of Agricultural and Food Chemistry*.

[43] Sharma M., Sandhir R., Singh A. (2016). Comparison analysis of phenolic compound characterization and their biosynthesis genes between two diverse bread wheat *(Triticum aestivum)* varieties differing for chapatti (unleavened flat bread) quality. *Frontiers in Plant Science*.

[44] Yang S. T., Wu X., Rui W., Guo J., Feng Y. E. (2015). UPLC/Q-TOF-MS analysis for identification of hydrophilic phenolics and lipophilic diterpenoids from radix salviae miltiorrhizae. *Acta Chromatographica*.

[45] Sun L., Tao S., Zhang S. (2019). Characterization and quantification of polyphenols and triterpenoids in thinned young fruits of ten pear varieties by UPLC-Q TRAP-MS/MS. *Molecules*.

[46] Kim J., Kim J., Lee C. W. (2016). Development and validation of a modified QuEChERS method coupled with LC-MS/MS to determine arbutin in pear peels. *Food Science and Biotechnology*.

[47] Hu F., An J., Li W. (2015). UPLC-MS/MS determination and gender-related pharmacokinetic study of five active ingredients in rat plasma after oral administration of *Eucommia cortex* extract. *Journal of Ethnopharmacology*.

[48] Chen W., Gong L., Guo Z. (2013). A novel integrated method for large-scale detection, identification, and quantification of widely targeted metabolites: application in the study of rice metabolomics. *Molecular Plant*.

[49] Sokolov S. Y., Ivashin V. M., Zapesochnaya G. G., Kurkin V. A., Shchavlinskii A. N. (1985). Studies of neurotropic activity of new compounds isolated from *Rhodiola rosea*. *Pharmaceutical Chemistry Journal*.

[50] Gryszczyńska A., Krajewska‐Patan A., Buchwald W. (2012). Comparison of proanthocyanidins content in *Rhodiola kirilowii* and *Rhodiola rosea* roots‐application of UPLC‐MS/MS method. *Herba Polonica*.

[51] Cirlini M., Mena P., Tassotti M. (2016). Phenolic and volatile composition of a dry spearmint (Mentha spicata L.). *Molecules*.

[52] Lee M. W., Lee Y. A., Park H. M. (2000). Antioxidative phenolic compounds from the roots of *Rhodiola sachalinensis* A. Bor. *Archives of Pharmacal Research*.

[53] Nakamura S., Li X., Matsuda H. (2007). Bioactive constituents from Chinese natural medicines. XXVI. Chemical structures and hepatoprotective effects of constituents from roots of *Rhodiola sachalinensis*. *Chemical and Pharmaceutical Bulletin*.

[54] Nakamura S., Li X., Matsuda H., Yoshikawa M. (2008). Bioactive constituents from Chinese natural medicines. XXVIII. Chemical structures of acyclic alcohol glycosides from the roots of *Rhodiola crenulata*. *Chemical and Pharmaceutical Bulletin*.

[55] Daikonya A., Kitanaka S. (2011). Constituents isolated from the roots of *Rhodiola sacra* S. H. Fu. Japan. *Journal of Food Chemistry Safety*.

[56] Viera M. N., Winterhalter P., Jerz G. (2016). Flavonoids from the flowers of *Impatients glandulifera* Royle isolated by high performance countercurrent chromatography. *Phytochemical Analysis*.

[57] Wang F., Li D., Han Z., Gao H., Wu L. (2007). Chemical constituents of *Rhodiola rosea* and inhibitory effect on UV‐induced A375‐S2 cell death. *Journal of Shenyang Pharmaceutical University*.

[58] Liu Q., Liu Z. L., Tian X. (2008). Phenolic components from *Rhodiola dumulosa*. *China Journal of Chinese Materia Medica*.

[59] Ni F., Xie X., Liu L. (2016). Flavonoids from roots and rhizomes of *Rhodiola crenulata*. *Chinese Traditional and Herbal Drugs*.

[60] Jeong H. J., Ryu Y. B., Park S. J. (2009). Neuraminidase inhibitory activities of flavonols isolated from *Rhodiola rosea* roots and their in vitro anti‐influenza viral activities. *Bioorganic & Medicinal Chemistry*.

[61] Ma C., Hu L., Kou X., Lv W., Lou Z., Wang H. (2017). Rapid screening of potential *α*-amylase inhibitors from *Rhodiola rosea* by UPLC-DAD-TOF-MS/MS-based metabolomic method. *Journal of Functional Foods*.

[62] Petsalo A., Jalonen J., Tolonen A. (2006). Identification of flavonoids of *Rhodiola rosea* by liquid chromatography‐tandem mass spectrometry. *Journal of Chromatography A*.

[63] Voskoboinikova I. V., Tjukavkina N. A., Geodakyan S. V. (1993). Experimental pharmacokinetics of biologically active plant phenolic compounds III. Pharmacokinetics of dihydroquercetin. *Phytotherapy Research*.

[64] Luo D., Zhao X., Wang J. (2005). Studies on the chemical constituents from *Rhodiola dumulosa* (I). *China Journal of Chinese Materia Medica*.

[65] Suzuki H., Sasaki R., Ogata Y. (2008). Metabolic profiling of flavonoids in *Lotus japonicus* using liquid chromatography Fourier transform ion cyclotron resonance mass spectrometry. *Phytochemistry*.

[66] Zhang S., Liu C., Bi H., Wang C. (2008). Extraction of flavonoids from *Rhodiola sachalinensis* A. Bor by UPE and the antioxidant activity of its extract. *Natural Product Research*.

[67] Wu S., Guo Y., Guo S., Li L., Wang B., Ma T. (2008). Study of the chemical constituents of ethanol extracts of *Rhodiola crenulata*. *Modern Food Science and Technology*.

[68] Choe K. I., Kwon J. H., Park K. H. (2012). The antioxidant and anti‐inflammatory effects of phenolic compounds isolated from the root of *Rhodiola sachalinensis* A. Bor. *Molecules*.

[69] Huang H., Liang M., Jiang P., Li Y., Zhang W., Gong Q. (2008). Quality evaluation of *Rhodiola crenulata*: quantitative and qualitative analysis of ten main components by HPLC. *Journal of Liquid Chromatography & Related Technologies*.

[70] Kurkin V. A., Zapesochnaya G. G., Nukhimovskii E. L., Klimakhin G. I. (1988). Chemical composition of rhizomes of a Mongolian *Rhodiola rosea* L. from districts near Moscow. *Pharmaceutical Chemistry Journal*.

[71] Satsyperova I. F., Pautova I. A., Kurkin V. A., Zapesochnaya G. G. (1993). Biologically active substances in rhizomes of *Rhodiola rosea* L. introduced in Petersburg. *Vegetable Resources*.

[72] Zuo G., Li Z., Chen L., Xu X. (2007). Activity of compounds from Chinese herbal medicine *Rhodiola kirilowii* (Regel) maxim against HCV NS3 serine protease. *Antiviral Reserach*.

[73] Sun J., Liang F., Bin Y., Li P., Duan C. (2007). Screening non-colored phenolics in red wines using liquid chromatography/ultraviolet and mass spectrometry/mass spectrometry libraries. *Molecules*.

[74] De Freitas M. A., Alves A. I. S., Andrade J. C. (2019). Evaluation of the antifungal activity of the *licania rigida* leaf ethanolic extract against biofilms formed by *Candida* sp. Isolates in acrylic resin discs. *Antibiotics*.

[75] Chu Y. H., Wu S. H., Hsieh J. F. (2014). Isolation and characterization of *α*‐glucosidase inhibitory constituents from *Rhodiola crenulata*. *Food Research International*.

[76] Chen L., Yu B., Zhang Y. (2015). Bioactivity‐guided fractionation of an antidiarrheal Chinese herb *Rhodiola kirilowii* (Regel) maxim reveals (−)‐epicatechin‐3‐gallate and (−)‐epigallocatechin‐3‐gallate as inhibitors of cystic fibrosis transmembrane conductance regulator. *PLoS One*.

[77] Wojakowska A., Perkowski J., Goral T., Stobiecki M. (2013). Structural characterization of flavonoid glycosides from leaves of wheat (*Triticum aestivum* L.) using LC/MS/MS profiling of the target compounds. *Journal of Mass Spectrometry*.

[78] Dinelli G., Segura-Carretero A., Di Silvestro R. (2011). Profiles of phenolic compounds in modern and old common wheat varieties determined by liquid chromatography coupled with time-of-flight mass spectrometry. *Journal of Chromatography A*.

[79] Kurkin V. A., Zapesochnaya G. G., Klyaznika V. G. (1982). Flavonoids of the rhizomes of *Rhodiola rosea*. I. Tricin glucosides. *Chemistry of Natural Compounds*.

[80] Mudge E., Lopes‐Lutz D., Brown P. N., Schieber A. (2013). Purification of phenylalkanoids and monoterpene glycosides from *Rhodiola rosea* L. roots by high‐speed counter‐current chromatography. *Phytochemical Analysis*.

[81] Furmanowa M., Skopińska‐Rozewska E., Rogala E., Hartwich M. (1998). Rhodiola rosea in vitro culture‐phytochemical analysis and antioxidant action. *Polish Botanical Journal*.

[82] Chen D., Fan J., Wang P. (2012). Isolation, identification and antioxidative capacity of water‐soluble phenylpropanoid compounds from *Rhodiola crenulata*. *Food Chemistry*.

[83] Ali Z., Fronczek F. R., Khan I. A. (2008). Phenylalkanoids and monoterpene analogues from the roots of *Rhodiola rosea*. *Planta Medica*.

[84] Yang Y., Feng Z., Jiang J., Zhang P. (2013). Chemical constituents of roots of Rhodiola crenulata. *Chinese Pharmaceutical Journal*.

[85] Krasnov E. A., Duvidzon L. M., Khnykina L. A., Evstigneeva R. P. (1966). Stimulators of the central nervous system.

[86] Diretto G., Jin X., Capell T., Zhu C., Gomez-Gomez L. (2019). Differential accumulation of pelargonidin glycosides in petals at three different developmental stages of the orange-flowered gentian (*Gentiana lutea* L. var. *aurantiac*a). *PLoS One*.

[87] Gard M., Chawla M., Chunduri V. (2016). Transfer of grain colors to elite wheat cultivars and their characterization. *Journal of Cereal Science*.

[88] Wang H., Xiao B., Hao Z., Sun Z. (2016). Simultaneous determination of fraxin and its metabolite, fraxetin, in rat plasma by liquid chromatography-tandem mass spectrometry and its application in a pharmacokinetic study. *Journal of Chromatography B*.

[89] Dinelli G., Marotti I., Bosi S. (2007). Lignan profile in seeds of modern and old Italian soft wheat (*Triticum aestivum* L.) cultivars as revealed by CE-MS analyses. *Electrophoresis*.

[90] Maldini M., Montoro P., Piacente S., Pizza C. (2009). Phenolic compounds from *Bursera simaruba* Sarg. bark: phytochemical investigation and quantitative analysis by tandem mass spectrometry. *Phytochemistry*.

[91] Kim J., Park Y. J., Park S. U., Ha S.-H., Kim J. K. (2018). Determination and quantification of arbutin in plants using stable isotope dilution liquid chromatography–mass spectrometry. *Applied Biological Chemistry*.

[92] Troshchenko A. T., Kutikova G. A. (1967). Rhodioloside from *Rhodiola rosea* and *R. quadrifida* I. *Chemistry of Natural Compounds*.

[93] Lihn P. T., Kim Y. H., Hong S. P., Jian J. J., Kang S. (2000). Quantitative determination of salidroside and tyrosol from the underground part of *Rhodiola rosea* by high performance liquid chromatography. *Archives of Pharmacal Research*.

[94] Golovina L. A., Nikonov G. K. (1988). Chemical study of *Rhodiola viridula*. *A Bor Isv Akad Nauk Kazak SSR Ser Khim*.

[95] Yoshikawa M., Nakamura S., Li X., Matsuda H. (2008). Reinvestigation of absolute stereostructure of (−)‐rosiridol: structures of monoterpene glycosides, rosiridin, rosiridosides A, B, and C, from Rhodiola sachalinensis. *Chemical and Pharmaceutical Bulletin*.

[96] Lee S.-Y., Choi Y.-J., Lee K.-B. (2008). Determination and monitoring of grayanotoxins in honey using LC-MS/MS. *Korean Journal of Food Science and Technology*.

[97] Peng J., Ma C., Ge Y. (2008). Chemical constituents and anti‐tuberculosis activity of root of *Rhodiola kirilowii*. *China Journal of Chinese Materia Medica*.

[98] Yoshikawa M., Shimada H., Horikawa S. (1997). Bioactive constituents of Chinese natural medicines. IV. Rhodiolae radix. (2): on the histamine release inhibitors from the underground part of Rhodiola sacra (PRAIN ex HAMET) S. H. Fu (Crassulaceae): chemical structures of rhodiocyanoside D and sacranosides A and B. *Chemical and Pharmaceutical Bulletin*.

